# Supplementary material for: In vitro toxicity and efficacy of verdinexor, an exportin 1 inhibitor, on opportunistic viruses affecting immunocompromised individuals
Source: PLoS One. 2018 Oct 17;13(10):e0200043. doi: 10.1371/journal.pone.0200043 (PMC6192554; doi:10.1371/journal.pone.0200043)
Supplement: S1 Table — (DOCX) [file pone.0200043.s001.docx]

| **Virus** | **Technique** | **Forward Primer** | **Reverse Primer** | **Probe** |
| --- | --- | --- | --- | --- |
| EBV | DNA hybridization | 5′-CCCAGGAGTCCCAGTAGTCA-3′ | 5′-CAGTTCCTCGCCTTAGGTTG-3′ |  |
| EBV | qPCR | 5′-CGGAAGCCCTCTGGACTTC-3′ | 5′-CCCTGTTTATCCGATGGAATG-3′ | 6FAM-TGTACACGCACGAGAAATGCGCC-TAMRA |
| KSHV | qPCR | 5′-TTCCCCAGATACACGACAGAATC-3 | 5′-CGGAGCGCAGGCTACCT-3′ | 5′-(6-carboxyfluorescein)-CCTACGTGTTCGTCGAC-(6-carboxytetramethylrhodamine)-3′ |
| HPV-11 | qPCR | 5′-TACGCCAGCTGGCGAAAGG-3′ | 5′-GCTTTACACTTTATGCTTCCGG-3′ | Sybr Green |
| HPV-18 | qPCR | 5′-AAGCTCAGCAGACGACCTTC-3′ | 5′-ACCTTCTGGATCAGCCATTG-3′ | Sybr Green |
| BKV | qPCR | 5’- AGTGGATGGGCAGCCTATGTA-3’ | 5’- TCATATCTGGGTCCCCTGGA-3’ | 5’-6-FAM-AGGTAGAAGAGGTTAGGGTGTTTGATGGCACAG-TAMRA-3’ |
| JCV | qPCR | 5’-CTGGTCATGTGGATGCTGTCA-3’ | 5’-GCCAGCAGGCTGTTGATACTG-3’ | 5’-6-FAM-CCCTTTGTTTGGCTGCT-TAMRA-3’ |
