# Supplementary figures and images for: In vitro toxicity and efficacy of verdinexor, an exportin 1 inhibitor, on opportunistic viruses affecting immunocompromised individuals
Source: PLoS One. 2018 Oct 17;13(10):e0200043. doi: 10.1371/journal.pone.0200043 (PMC6192554; doi:10.1371/journal.pone.0200043)

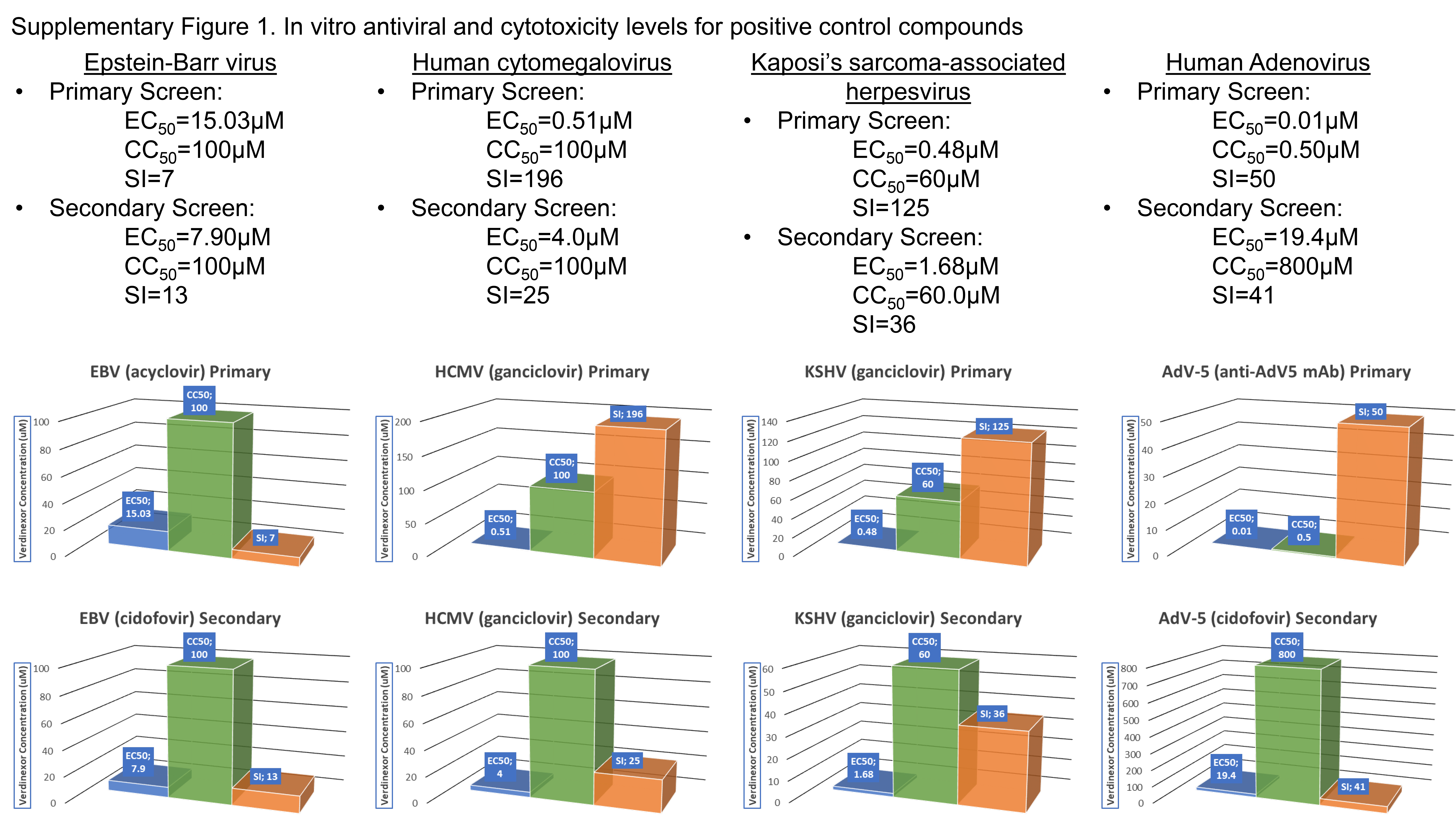

Supplement: S1 Fig — Results of positive control treatment against viral infections. EC50 values are plotted in blue, CC50 values are plotted in green, and the SI value for each assay is plotted in orange. (TIF) [file pone.0200043.s002.tif]

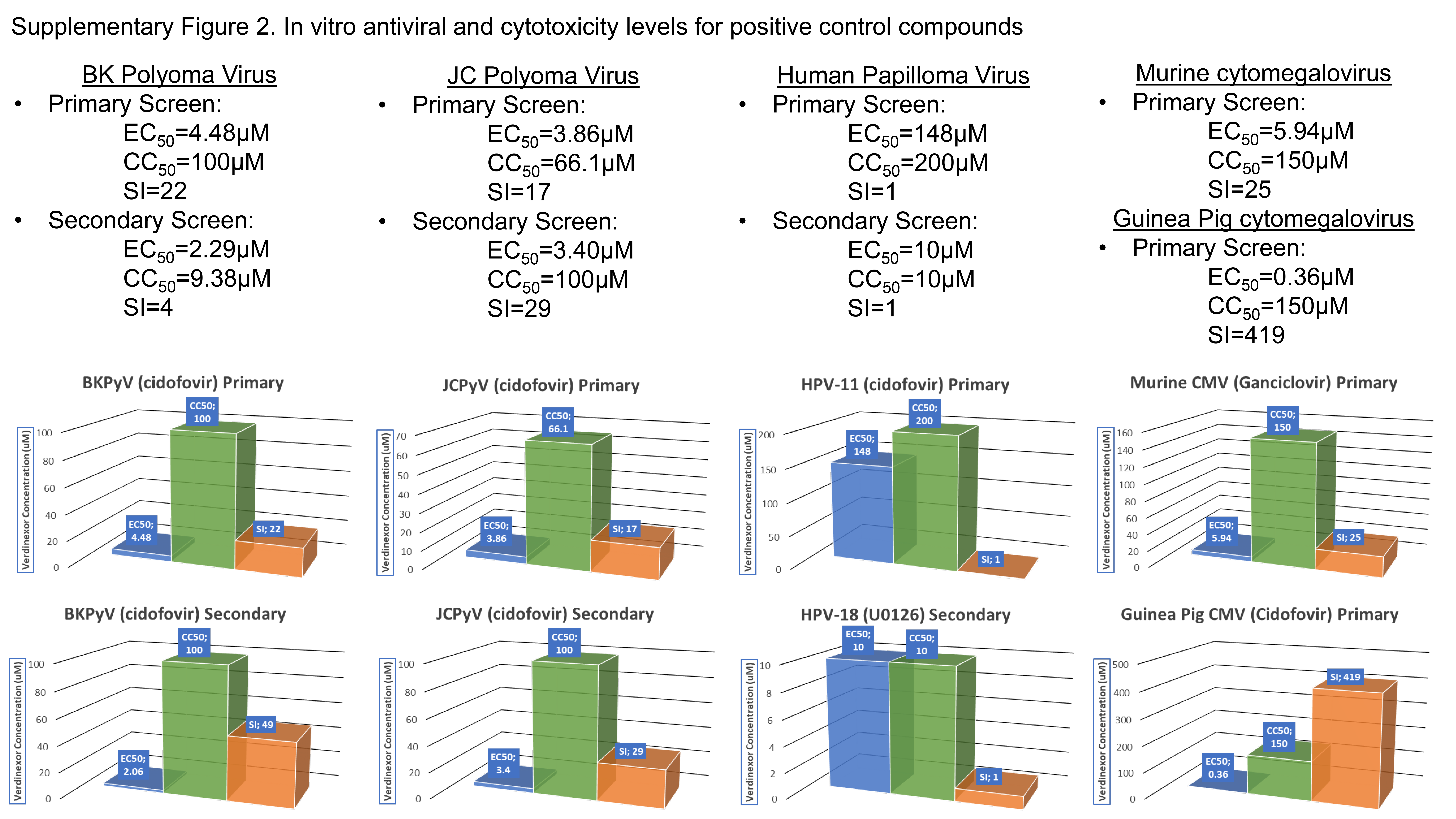

Supplement: S2 Fig — Results of positive control treatment against viral infections. EC50 values are plotted in blue, CC50 values are plotted in green, and the SI value for each assay is plotted in orange. (TIF) [file pone.0200043.s003.tif]
